# Seroprevalence study of hepatitis A virus in Fars province, southern Iran

**Published:** 2011-04-01

**Authors:** Seyed Alireza Taghavi, Mohammad Kazem Hosseini Asl, Mozaffar Talebzadeh, Ahad Eshraghian

**Affiliations:** 1 Department of Medicine, Shiraz University of Medical Science, Gastroenterology Research Center, Nemazi Hospital, Shiraz, IR Iran

**Keywords:** Hepatitis A virus, Seroprevalence, Iran, Epidemiology

## Abstract

**Background:**

There are several studies on seroprevalence of hepatitis A virus (HAV) in adults in the Middle East.

**Objectives:**

To determine seroprevalence of HAV among adult population in Fars province, southern Iran.

**Patients and Methods:**

In a cross-sectional study, we checked anti-HAV antibody (IgG) in subjects refereed to our health care centers to perform laboratory tests before getting married between March 2008 and March 2009. Age-specific seroprevalence was also determined. Some risk factors like level of education, type of residence, job, numbers of family members, and access to treated water were also evaluated in these participants.

**Results:**

From 1050 subjects studied, 927 (88.2%) had ant-HAV antibody; 123 (11.8%) were antibody negative. Among subjects aged < 20 years, the anti-HAV seroprevalence was the lowest (79.3%) followed by subjects aged 20-30 years (91.3%) and those > 30 years (99%) (p = 0.01). 85.1% of studied individuals in urban areas had anti-HAV IgG while 95.9% of subjects in rural regions were anti-HAV positive (p = 0.001). The seroprevalence of HAV antibody was significantly associated with number of family members (p = 0.001).

**Conclusion:**

HAV is highly prevalent in our region especially in rural areas. It is better to vaccinate the children for HAV by the time they receive HBV vaccine or when they are five years.

## Background

Hepatitis A virus (HAV) is a RNA virus belonging to the family Picornaviridae and is the most common cause of acute viral hepatitis worldwide [1]. The virus is transmitted via orofecal route predominantly by ingestion of infected food and water or direct contact with an infected person [1]. This virus is more prevalent in low socioeconomic societies, crowded regions and those using untreated water. Previously, most people were infected with HAV in their early childhood, spend an uncomplicated disease with minimal symptoms and 90 % of them have acquired natural immunity to this pathogen for the rest of their life. During recent decades and in parallel to improvement in health care systems among developing countries, the pattern of this viral infection has shifted from childhood to adolescence with more severe and even life-threatening course [2]. Since lots of infected individuals have mild symptoms or even remain asymptomatic in the course of infection, epidemiological features are described by serological tests [3]. Three epidemiological patterns of endemicity are observed throughout the world and are divided into "low," "intermediate" and "high" and are dependent to age and level of hygiene [4]. Several studies were conducted on the prevalence of HAV in Iranian children and adults, however, more studies seems to be required for a better understanding of the disease and to design preventive strategies in different country regions. The seroprevalence of HAV was 61.5% in children of Tehran and was increased in older ages [5]. Another study in Northeast of Iran showed a seroprevalence rate of 86.8% among young adults [6][7]. Vaccination against HAV is not recommended routinely among patients in childhood based on its benign and uncomplicated course. However, recommendations for adult vaccination remains to be elucidated in epidemiological studies investigating the immunity of adults against this virus.

## Objectives

This study was conducted to evaluate the seroprevalence and risk factors of HAV virus in Fars province, southern Iran.

## Patients and Methods

### Study population

Fars province, with 4.4 million population in 2007, is one of the largest provinces of Iran located in the South and Southwest of Iran. Shiraz with a population of nearly 1.8 million is the capital of Fars province and one of the Iranian metropolises. Besides Shiraz, two northern cities of Fars province were also selected for sampling. Individuals were selected among those referred to our health care centers to perform screening laboratory tests before getting married between March 2008 and March 2009. All of these participants were Iranian and permanent inhabitants of the selected areas. These subjects were from all socioeconomic classes and among adults from both sexes and thus could reflect the general adult population of the society. Those who refused to participate were excluded from the study. Table 1 shows the demographic information of Iran, Fars and the three studied cities. Based on heterogeneity in the reported prevalence rates of HAV in previous studies, considering two genders and three age groups, the study sample size was estimated to be 1050 subjects. Sampling was made using a multi-stage random sampling method.

**Table 1:** Census data of Iran, Fars and the three studied cities (Source: Iran National Population and Housing Census, 2006)

-	**Area **(km(2))	**Population**	**Percent urban**	**Percent mail**
**Iran**	1,648,195	70,495,782	68.5%	50.9%
**Fras**	133100	4,336,878	61.17%	50.8%
**Shiraz**	10688	1,711,186	77.37%	51.3%
**Mamasani**	6800	166,308	34.8%	49.5%
**Abadeh**	6052	92,959	89.4%	51.3%

### Data collection

Using a questionnaire, epidemiological data including age, gender, type of residence (rural vs urban area), place of residence, level of education, job, number of family members and access to adequate treated water were collected. Blood samples were obtained from all individuals referring to our health care centers for screening laboratory tests before marriage. All samples required for this study transferred to Gastroenterology and Hepatology Research Center affiliated to Shiraz University of Medical Science, where they were tested by experienced technicians. Serum total anti-HAV (IgG) antibody levels were determined using DiaPro kits (Diagnostic Bioprobes srl, Milano, Italy).

### Ethics and consent

The study protocol was confirmed by Ethical Committee of Shiraz University of Medical Science. The protocol and goals of the study were described for participants and each gave informed written consent. The study was conducted in accordance with the Helsinki declaration (Edinburgh revision, 2000).

### Statistical analysis

All data were expressed as mean ± SD. Categorical variables were compared by x(2), and correlation analyses were performed using Pearson's correlation coefficient. A p < 0.05 was considered statistically significant. Statistical analyses were performed using SPSS® 11 software for Windows® (SPSS, Inc., Chicago, IL, USA).

## Results

Totally, 1050 subjects were enrolled in this study. There were 523 (49.8%) males and 527 (50.2%) females. Eight-hundred and seventy (82.8%) participants were from Shiraz, 120 (11.5%) from Mamasani, and 60 (5.7%) from Abadeh. From 1050 studied subjects, 927 (88.2%) had antibody against HAV and 123 (11.8%) were antibody negative. Seroprevalence of HAV antibody in the participants, according to the city of residence is outlined in Table 2.

**Table 2:** Seroprevalence of HAV antibody in the participants

** City **	** Negative **No. (%)	** Positive **No. (%)
** Shiraz **	108 (12.4%)	762 (87.6%)
** Mamasani **	5 (4.1%)	115 (95.9%)
** Abadeh **	10 (16.7%)	50 (83.3%)
** Total **	123 (11.8%)	927 (88.2%)

The mean age of participants was 25 (range: 15-63) years. Participants were categorized into three age groups: < 20, 20-30, and >30 years. Age-specific seroprevalence of anti-HAV antibody is shown in Table 3.

**Table 3:** Age-specific seroprevalence of anti-HAV antibody in studied subjects

** Age group **	** Negative **No. (%)	** Positive** No. (%)
** < 20 **	67 (20.7%)	256 (79.3%)
** 20–30 **	55 (8.7%)	572 (91.3%)
** > 30 **	1 (1%)	99 (99%)

Among subjects aged < 20 years, the anti-HAV seroprevalence was the lowest (79.3%); it followed by subjects aged 20-30 years (91.3%), and those > 30 years (99%) (p = 0.01). Table 4 shows sex-specific distribution of anti-HAV antibody among our patients. According to the level of education, participants were categorized as follows: 1% of the study population were "uneducated;" 17.7% of subjects had a "preliminary education;" 2.8% completed third year of high school; 55.1% of subjects had high school diploma and 23.4% had continued their education after diploma. Seroprevalence of anti-HAV antibody according to the level of education is shown in Table 4.

**Table 4:** Seroprevalence of anti-HAV antibody according to sex, level of education, type of residence, jobs, water access and numbers of family member

	**Negative **No. (%)	**Positive **No. (%)
** Sex **		
** Male **	44 (8.4%)	479 (91.6%)
** Female **	79 (14.9%)	448 (85.1%)
** Education **		
** Uneducated **	0 (0%)	10 (100%)
** Preliminary **	13 (7.2%)	169 (92.8%)
** High school **	3 (10.3%)	260 (89.7%)
** Diploma **	70 (11.9%)	518 (88.1%)
** Post-diploma **	30 (12.5%)	211 (87.5%)
** Type of Residence **		
** Urbant **	109 (14.9%)	620 (85.1%)
** Rural **	13 (4.1%)	308 (95.9%)
**Job **		
** Unemployed/Household **	55 (14.8%)	316 (85.2%)
** Worker **	3 (3.85%)	77 (96.2%)
** Clerk **	16 (11.6%)	122 (88.4%)
** Others **	32 (6.9%)	429 (93.1%)
**Treated water **		
** Yes **	120 (11.8%)	892 (88.2%)
** No **	2 (5.2%)	36 (94.8%)
** Family members **		
** < 4 **	33 (19.9%)	133 (80.1%)
** 4–6 **	51 (11.6%)	386 (88.4%)
** > 6 **	34 (7.6%)	413 (92.4%)

All of uneducated individuals were found anti-HAV positive. However, there was no statistically significant different in anti-HAV seroprevalence among participants with different levels of education (p = 0.25). Seven-hundred and twenty-nine (69%) of subjects resided in urban area while 321 (31%) were from rural areas (Table 4). Eighty-five and one-tenth percent of individuals in urban areas had anti-HAV IgG while 95.9% of subjects in rural regions were anti-HAV IgG positive (p = 0.001). Ninety-six and three-tenth percent of subjects had access to adequate treated water while 3.7% used untreated water (Table 4). Seroprevalence of anti-HAV antibody stratified by the number of family members is shown in Table 4-the higher the number of family members, the higher the seroprevalence of HAV (p = 0.001).

## Discussion

In this cross-sectional study, we investigated the seroprevalence of HAV among adults from three cities of Fars province, southern Iran. HAV is highly prevalent in Fars province. We found an overall seroprevalence of 88.2% in our population which is nearly similar to reports from other parts of Iran [8][9]. Among these cities, Mamasani had the highest prevalence of HAV antibody (95.9%) that can be justified by the higher percentage of rural population in this city compared to the two other cities. This pattern preserved in Shiraz and Abadeh, that is, Abadeh with the lowest rural population had the lowest HAV seroprevalence. As expected, the prevalence of HAV was higher in old ages; the rate reached to 99% in participants > 30 years. We also determined the seroprevalence of HAV in five groups of education. The serprevalence of HAV antibody was lower in participants with higher educations (Table 4); the rate was 100% in uneducated peoples. Older age, residing in rural areas, numbers of family members were risk factors for HAV infection. One Iranian study reported a higher prevalence of HAV among older subjects and in urban areas [9]. The overall seroprevalence of HAV was 86% in this study. The seroprevalence of HAV was 22.3% among children in Tehran (capital of Iran) hospitals. This study reported gradual rise in HAV seroprevalence in older ages [10]. Another study showed that the seroprevalence of HAV in patients with chronic hepatitis B infection was 82.1%, which is similar to healthy population [11]. A cross-sectional study conducted in Bangladesh reported a seroprvalence rate of 69.6 % in Bangladeshi population. Older ages, rural areas and lower socioeconomic status were risk factors for HAV infection [12]. Our finding about association of age with seroprevalence of HAV antibody is concordant with results of other studies in developing countries, especially Asian countries [13][14]. Other studies in Asian population also showed a higher prevalence of HAV antibody in rural regions compared to urban areas [15][16][17]. Urban populations usually have better access to treated water and other sanitary services, higher socioeconomic status and lower number of family members.

In conclusion, according to results of this study, vaccination against HAV may be helpful in adults in younger age groups and people from urban areas. However, it is not justified in rural areas since most of adults were infected during childhood and are immunized against HAV. More studies on cost-effectiveness and other aspects of HAV vaccination must be conducted for better assessment of its benefits.
